# A hybrid method to improve efficiency of patient specific SRS and SBRT QA using 3D secondary dose verification

**DOI:** 10.1002/acm2.13858

**Published:** 2022-12-30

**Authors:** Garrett C. Baltz, Remy Manigold, Richard Seier, Steven M. Kirsner

**Affiliations:** ^1^ Scripps MD Anderson Cancer Center San Diego California USA

**Keywords:** IMRT QA, MU verification, patient specific quality assurance (QA), SBRT, secondary dose verification, SRS, workflow

## Abstract

**Purpose:**

Patient Specific QA (PSQA) by direct phantom measurement for all intensity modulated radiation therapy (IMRT) cases is labor intensive and an inefficient use of the Medical Physicist's time. The purpose of this work was to develop a hybrid quality assurance (QA) technique utilizing 3D dose verification as a screening tool to determine if a measurement is necessary.

**Methods:**

This study utilized Sun Nuclear DoseCHECK (DC), a 3D secondary verification software, and Fraction 0, a trajectory log IMRT QA software. Twenty‐two Lung stereotactic body radiation therapy (SBRT) and thirty single isocentre multi‐lesion SRS (MLSRS) plans were retrospectively analysed in DC. Agreement of DC and the TPS dose for selected dosimetric criteria was recorded. Calculated 95% confidence limits (CL) were used to establish action limits. All cases were delivered and measured using the Sun Nuclear stereotactic radiosurgery (SRS) MapCheck. Trajectory logs of the delivery were used to calculate Fraction 0 results for the same criteria calculated by DC. Correlation of DC and Fraction 0 results were calculated. Phantom measured QA was compared to Fraction 0 QA results for the cases which had DC criteria action limits exceeded.

**Results:**

Correlation of DC and Fraction 0 results were excellent, demonstrating the same action limits could be used for both and DC can predict Fraction 0 results. Based on the calculated action limits, zero lung SBRT cases and six MLSRS cases were identified as requiring a measurement. All plans that passed the DC screening had a passing measurement based PSQA and agreed with Fraction 0 results.

**Conclusion:**

Using 95% CL action limits of dosimetric criteria, a 3D secondary dose verification can be used to determine if a measurement is required for PSQA. This method is efficient for it is part of the normal clinical workflow when verifying any clinical treatment. In addition, it can drastically reduce the number of measurements needed for PSQA.

## 1 INTRODUCTION

The independent secondary verification of monitor units (MU) for radiotherapy treatment plans is an important component of the process to verify the quality and safety of a plan.^1^, ^2^ While historically this verification is performed by comparing the calculated dose to a point, newer secondary check software is capable of independently calculating the full 3D dose distribution using treatment planning system (TPS) class dose algorithms.^3^, ^4^ Compared to conventional methods, 3D dose distributions allow for a more comprehensive verification of the treatment plan, including target coverage, dose volume histogram (DVH) comparisons, and 3D Gamma analysis.^5^

An equally important component of the quality assurance (QA) process for intensity modulated radiation therapy (IMRT) or volumetric modulated arc therapy (VMAT) plans is patient‐specific QA (PSQA) of the final treatment plan. These plans are highly complex due to combined modulation of dose rate, gantry speed, and the use of small multileaf collimator (MLC) apertures. The complexity of these plans and uncertainties in modelling the MLC, collimation penumbra, small field output factors, and off‐axis profiles led to the establishment of measurement‐based patient‐specific QA as a standard component of IMRT/VMAT plan QA.^6^ PSQA measurements provide verification that the dose distribution planned in the TPS can be delivered accurately by the machine used to treat the patient. It can be especially valuable for plans which heavily rely on delivering large doses with small apertures such as lung stereotactic body radiation therapy (SBRT) and single isocentre multi‐lesion brain stereotactic radiosurgery (SRS).

Popular measurement‐based methods for PSQA of lung SBRT plans include film,^7^, ^8^, ^9^ multi‐dimensional diode arrays,^10^, ^11^, ^12^ high‐resolution diode arrays,^10^, ^13^, ^14^ and electronic portal imaging device (EPID) based QA.^12^, ^15^, ^16^, ^17^, ^18^ Due to the use of small field segments and steep dose gradients that are associated with lung SBRT plans, previous studies have demonstrated the need to use high resolution measurement methods to be able to detect potential errors through the use of stricter gamma criteria.^10^, ^12^, ^17^ However, even with high resolution measurement devices, the sensitivity of measurement based QA to detect errors has been shown to be limited.^19^, ^20^, ^21^, ^22^ Similar to lung SBRT, single isocentre multi‐lesion SRS plans frequently make use of small field segments and high dose gradients. An additional challenge to performing PSQA for single isocentre multi‐lesion SRS plans is the geometric distribution of the multiple targets makes it difficult to perform measurement based PSQA for all targets. Increasingly multi‐lesion SRS plans can have a dozen or more lesions, and it is not practical to acquire a measurement for every lesion when SRS plans are often thousands of MU. Techniques presented in the literature have proposed measuring a single lesion,^10^ measuring the largest and the smallest lesion,^23^ or trying to measure at least three lesions on a single plane.^8^ Given the efficiency challenges associated with these techniques, there is motivation to develop a more efficient alternative method.

The advancement of independent secondary 3D dose verification software provides an opportunity for secondary dose calculations to serve a larger role in the PSQA process. While previously computation‐based verification of dose for IMRT/VMAT plans was limited, now independent 3D dose calculations can provide a more comprehensive verification of the TPS calculated dose. There are many commercial software solutions now available, including DoseCHECK (Sun Nuclear), Mobius 3D (Varian), and VERIQA (PTW). However, a critical component of any QA program is the existence of properly selected action limits for the quality metrics that dictate if a plan is clinically acceptable. Due to the relative novelty of 3D secondary dose verification, there have been a limited number of studies presenting typical agreement of 3D secondary verifications.^3^, ^24^, ^25^, ^26^, ^27^ The lack of an abundance of published data means there are not generally accepted criteria for the metrics unique to 3D secondary verification. This is demonstrated in the recent AAPM TG‐219 report in which no concrete recommendations could be made regarding criteria to be used for 3D verification.^28^

With the goal to advance the use of 3D secondary verification for PSQA and to reduce the amount of measurement‐based PSQA, the purpose of the current study was twofold: to first characterize the typical agreement of independent 3D dose verification software for small field stereotactic treatments to develop action limits, and second, to present an efficient hybrid technique for performing patient‐specific QA of stereotactic treatment plans.

## 2 METHODS

### 2.1 Proposed hybrid PSQA technique

The secondary dose/MU verification software used in the current study was Sun Nuclear (Melbourne, Florida) DoseCHECK (DC). DC uses the DICOM RT Plan, RT Structure Set, and patient CT dataset exported from the TPS to independently calculate the 3D dose distribution using the Sun Nuclear Dose Calculator (SDC). SDC is a convolution/superposition GPU‐accelerated dose calculation algorithm that has been fully benchmarked as a clinical TPS‐class dose algorithm.^4^ A complementary software called PerFRACTION uses the linear accelerator's trajectory log files recorded during plan delivery to reconstruct an RT Plan file of the plan delivery. SDC uses this reconstructed RT Plan to calculate the 3D delivered dose on the patient CT. PerFRACTION can be used for both phantom‐less pre‐treatment QA (Fraction 0) and for in‐vivo monitoring of every fraction delivery. Together, this software can provide a comprehensive computational‐based method of PSQA, which can reduce the need for measurement‐based QA and increase efficiency.

The proposed hybrid PSQA technique uses the 3D secondary dose calculation (DC) as an initial screening to determine which plans will require further measurement based PSQA. Action limits were set for PTV coverage metrics, point doses, and 3D Gamma results. All plans will be delivered before treatment, so Fraction 0 (F0) pre‐treatment QA can be performed. Plans for which any of the metrics exceed their established action limit for either the DC or F0 dose calculations will require an additional measurement based PSQA to be performed.

Two specific treatment sites that are historically difficult to verify with an independent calculation and commonly utilize traditional phantom based measurement techniques for PSQA were selected for evaluation of the proposed technique. These two sites were lung SBRT and single isocentre multi‐lesion brain SRS (MLSRS) plans.

### 2.2 Commissioning of the 3D secondary verification software

Initially, in collaboration with the vendor, Sun Nuclear, customized beam models were developed for seven beam matched Varian (Varian Medical Associates, Palo Alto, California) linear accelerators. This study used only the TrueBeams equipped with the high‐definition MLC. Models were created for both the AAA and Acuros XB (AXB) dose calculation algorithms utilized by the Varian Eclipse v15.6 TPS using the process previously described in the literature by Bismack et al.^27^ The finalized beam models were validated using methods described in AAPM TG‐53 and TG‐119.^29^, ^30^ In addition, they were verified with measurements using standard QA phantoms: the Sun Nuclear SRS MapCheck and ArcCheck, as well as dosimetric comparisons with over 100 clinically approved treatment plans.

### 2.3 Determination of action limits for 3D secondary verification

DC and F0 can be configured to report several metrics to quantify agreement with the TPS calculated dose for 3D independent verification. The primary metric is the overall 3D Gamma of the dose within the external contour. Target related metrics can be reported for the planning target volume (PTV) or internal target volume (ITV) including: Gamma within the volume, percentage difference of mean dose, max dose, and centroid point dose of the target. DVH based metrics such as D90 and D95 can also be reported. DC and F0 were configured to calculate the dose using the same dose grid resolution as the TPS.

The TG‐119 confidence limit formalism was selected for determination of action limits.^30^ Briefly summarized here: for a given metric, the average and standard deviation (SD) of the difference between DC and TPS was calculated, from which the 95% confidence limit (CL) can be calculated using Eq. (1).

(1) $$\mathrm{CL}=\left|\mathrm{average}\;\mathrm{difference}\right|+1.96\times \mathrm{SD}$$

#### 2.3.1 Lung SBRT

**FIGURE 1:**
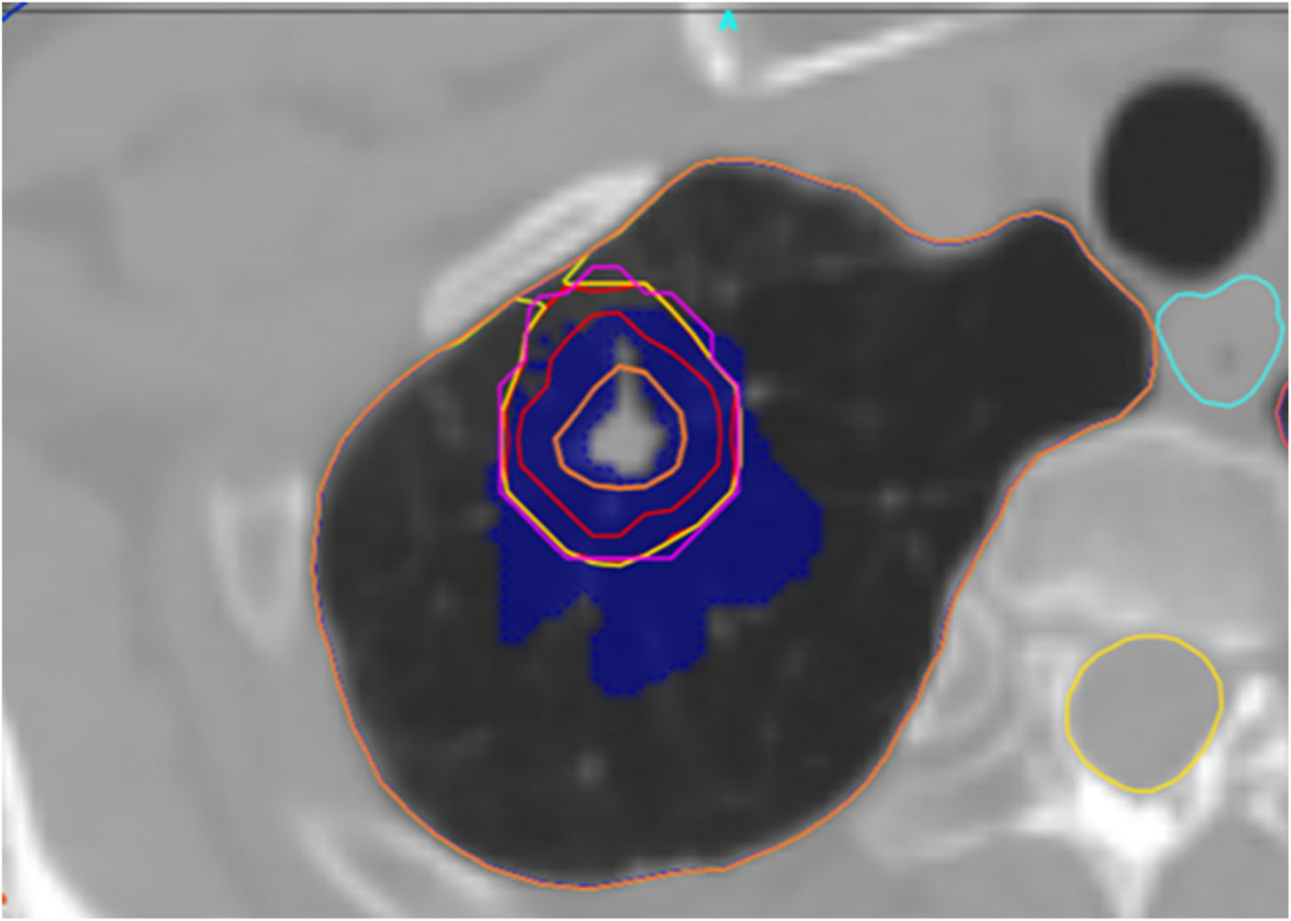
Example DC 3D gamma analysis for a lung SBRT case. Points failing cold are shown in blue and can be seen within the PTV contour (pink). Gamma pass rate (3%/1 mm) was 91% with a 10% threshold and 64% with a 40% threshold.

To establish criteria for Lung SBRT cases, 22 clinical plans were selected for retrospective analysis. All plans were generated in Eclipse v15.6 TPS and delivered 60 Gy to the ITV, 50 Gy to the PTV (5 mm expansion) in four fractions using two ipsilateral VMAT arcs and with a beam energy of 6XFFF. Planning CTs were either an average CT generated from a 4DCT or a breath hold CT, acquired with 120 kVp, 225 mAs, 65 cm field of view, and 1.25 mm slice thickness. The final dose was calculated using a 1.25 mm dose grid. Metrics selected for action limit determination were isocentre dose, mean and max dose to the PTV, PTV D90 and D95, as well as total 3D gamma. Gamma analysis criteria used 3% dose difference, 1 mm distance‐to‐agreement, global normalization with a 40% threshold. A 40% dose threshold was selected as preliminary investigation demonstrated much higher gamma pass rates when using a typical threshold of 10%, which has been previously reported in the literature regarding 3D Gamma.^31^ The authors attribute this mostly to the sheer number of points that are analysed in a 3D Gamma, up to 300 000 points compared to several hundred for a typical phantom QA. An example of how using a low threshold can be misleading is demonstrated in Figure 1, where a large portion of the PTV (pink contour) contains cold failing points shown in blue. Using 3%/1 mm and 10% threshold for this case, results in a passing rate of 91%. However, using a 40% threshold results in a passing rate of only 64%. A 40% threshold was chosen for the current study as a balance to increase sensitivity while still including a low enough dose level relevant to OAR planning constraints as part of the analysis.

DC results for all metrics were recorded and histogram plots were generated to determine mean percentage difference and the SD for the metric to calculate the confidence limit.

Preliminary investigation demonstrated significant difference in the agreement of DC to TPS depending on the TPS dose calculation algorithm used. Therefore, all plans had a final dose calculation in Eclipse using both the AAA and Acuros XB algorithms, and separate confidence limit analyses were performed.

#### 2.3.2 Multi‐lesion brain SRS

Criteria establishment for the single isocentre MLSRS cases utilized 30 clinical cases chosen retrospectively with a total of 82 individual targets. All plans were generated using the Brainlab (Munich, Germany) Elements Multiple Brain Mets SRS v3.0 TPS. Elements automatically inversely optimizes a dynamic conformal arc treatment plan using 3–12 half arcs distributed among 3–6 couch angles. The prescribed dose varied from 18 to 22 Gy to the PTV (1 mm expansion from GTV) and all plans were generated using 6XFFF, with dose calculated by the Brainlab Pencil Beam algorithm on a 1 mm dose grid. Planning CTs were acquired with 120 kVp, 440 mA, 40 cm field of view, and 1 mm slice thickness.

Metrics chosen for analysis of the MLSRS cases were mean and max dose to the PTV, dose to the centroid point of each PTV, PTV D90 and D95, and the total 3D gamma. Gamma analysis criteria used 3% dose difference, 1 mm distance‐to‐agreement, and global normalization with a 10% threshold.

As with the lung SBRT plans, DC results for all metrics were recorded and histogram plots were generated for the calculation of the confidence limits.

### 2.4 Validation of hybrid PSQA technique

All plans for both the lung SBRT and MLSRS cases were delivered and measured using the Sun Nuclear SRS MapCheck in the Stereophan. Gamma analysis used 3% dose difference, 1 mm distance to agreement, global normalization with a 10% threshold. Trajectory logs of the QA delivery were obtained and used to calculate the Fraction 0 PSQA.

#### 2.4.1 Correlation of DC and Fraction 0

To determine whether the DC or Fraction 0 calculation should be used as the screening, correlation of the results for DC and Fraction 0 were performed for all criteria discussed earlier for the MLSRS as well as the lung SBRT cases. Correlation coefficients were calculated for each of the criteria from plots of the results of DC versus Fraction 0. The correlation coefficient was used to determine if DC could be used as a predictor for the Fraction 0 results, and if the same action limits can be used for both.

#### 2.4.2 QA result comparison

Validation of the hybrid PSQA technique was tested by applying the respective calculated confidence limits to both the DC and F0 results for every plan. For any case that didn't achieve passing results for any chosen criteria, comparisons to the SRS MapCheck measurements were made to see if the phantom measured results showed similar or passing results.

## 3 RESULTS

### 3.1 Action limits for lung SBRT

**FIGURE 2:**
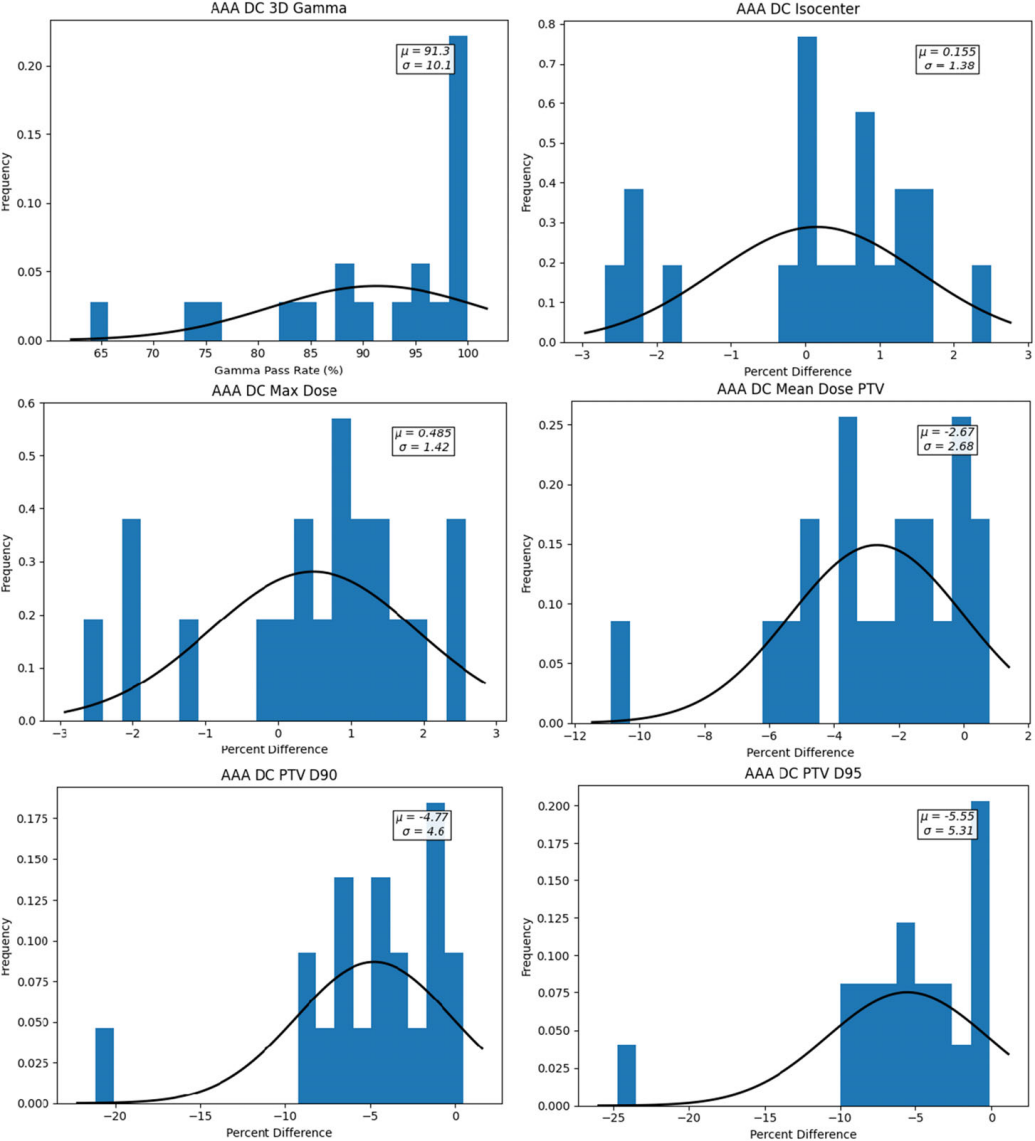
Histogram plots of DC dosimetric criteria agreement for the lung SBRT plans calculated with AAA

Figure 2 presents the histogram plots of the DC agreement of the chosen dosimetric criteria for the Lung SBRT plans calculated with the AAA dose algorithm.

Table 1 summarizes the average percentage difference (%Δ) and SD derived from the histogram plots for each of the criteria. The CL action limits calculated using Eq. (1) were: 3% for isocentre dose, 3% for maximum dose, 8% for the mean PTV dose, 14% for the PTV D90, and 16% for the PTV D95.

**TABLE 1:** Summary of the AAA lung SBRT DC dosimetric criteria results and corresponding calculated CL

Criteria	Average	SD	CL
Isocentre	0.16	1.38	3%
Max dose	0.49	1.42	3%
PTV mean dose	−2.67	2.68	8%
PTV D90	−4.77	4.6	14%
PTV D95	−5.55	5.31	16%
3D gamma	91.3	10.1	28.5(71.5%)

**FIGURE 3:**
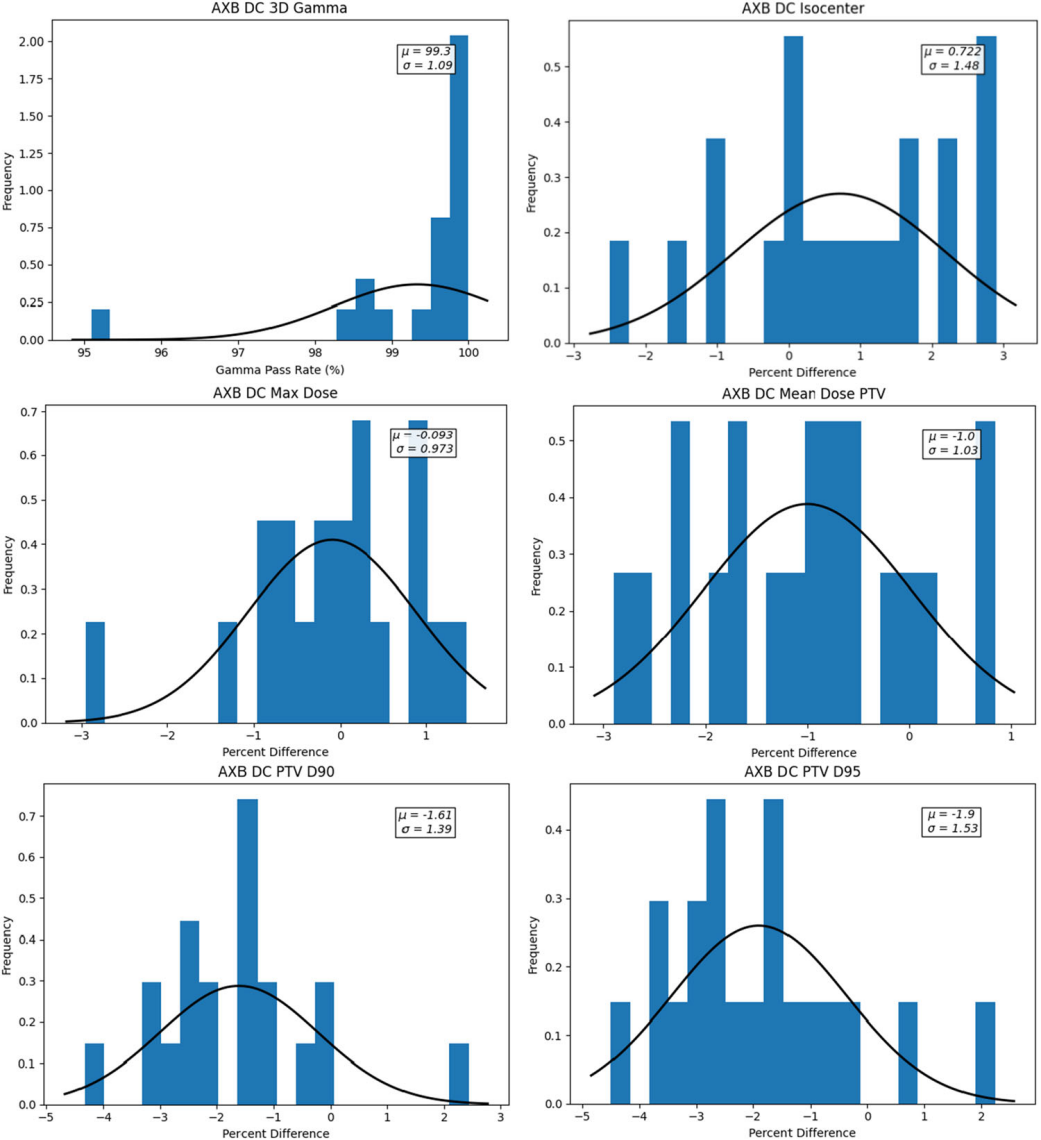
Histogram plots of DC dosimetric criteria agreement for the lung SBRT plans calculated with AXB

Figure 3 presents the histogram plots of the dosimetric criteria agreement for the Lung SBRT plans calculated with the Acuros XB dose algorithm.

Table 2 summarizes the Acuros XB DC agreement and the calculated CL action limits were: 4% for isocentre or centroid dose, 2% for maximum dose, 3% for the mean PTV dose, 4% for the PTV D90 and 5% for the PTV D95. To simplify analysis, 3% was chosen as the action limit for all criteria for plans calculated using the Acuros XB algorithm.

**TABLE 2:** Summary of the acuros XB lung SBRT DC dosimetric criteria results and corresponding calculated CL

Criteria	Average	SD	CL
Isocentre	0.72	1.48	4%
Max dose	−0.09	0.97	2%
PTV mean dose	−1.00	1.03	3%
PTV D90	−1.61	1.39	4%
PTV D95	−1.9	1.53	5%
3D gamma	99.3	1.09	2.8(97.2%)

The results demonstrate a significant difference in the calculated CL for the dosimetric criteria for plans calculated using AAA versus Acuros XB. Agreement of AAA and DC was much poorer compared to Acuros XB and DC.

### 3.2 Correlation of DC and Fraction 0 for lung SBRT

**FIGURE 4:**
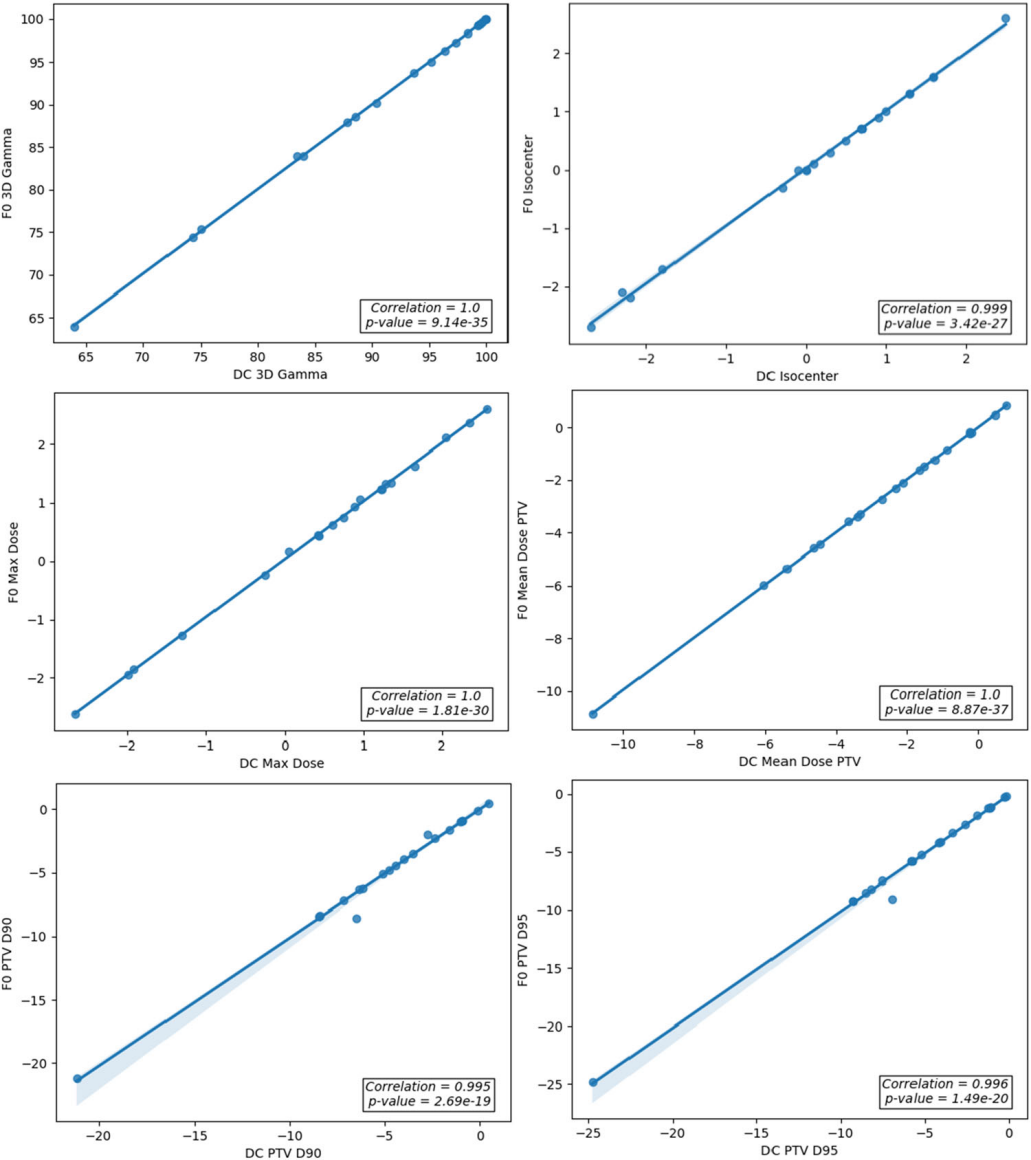
Correlation plots of dosimetric criteria calculated by DC and F0 for AAA lung SBRT plans

Figure 4 demonstrates the correlation between DC and F0 for treatment plans calculated with the AAA algorithm.

**FIGURE 5:**
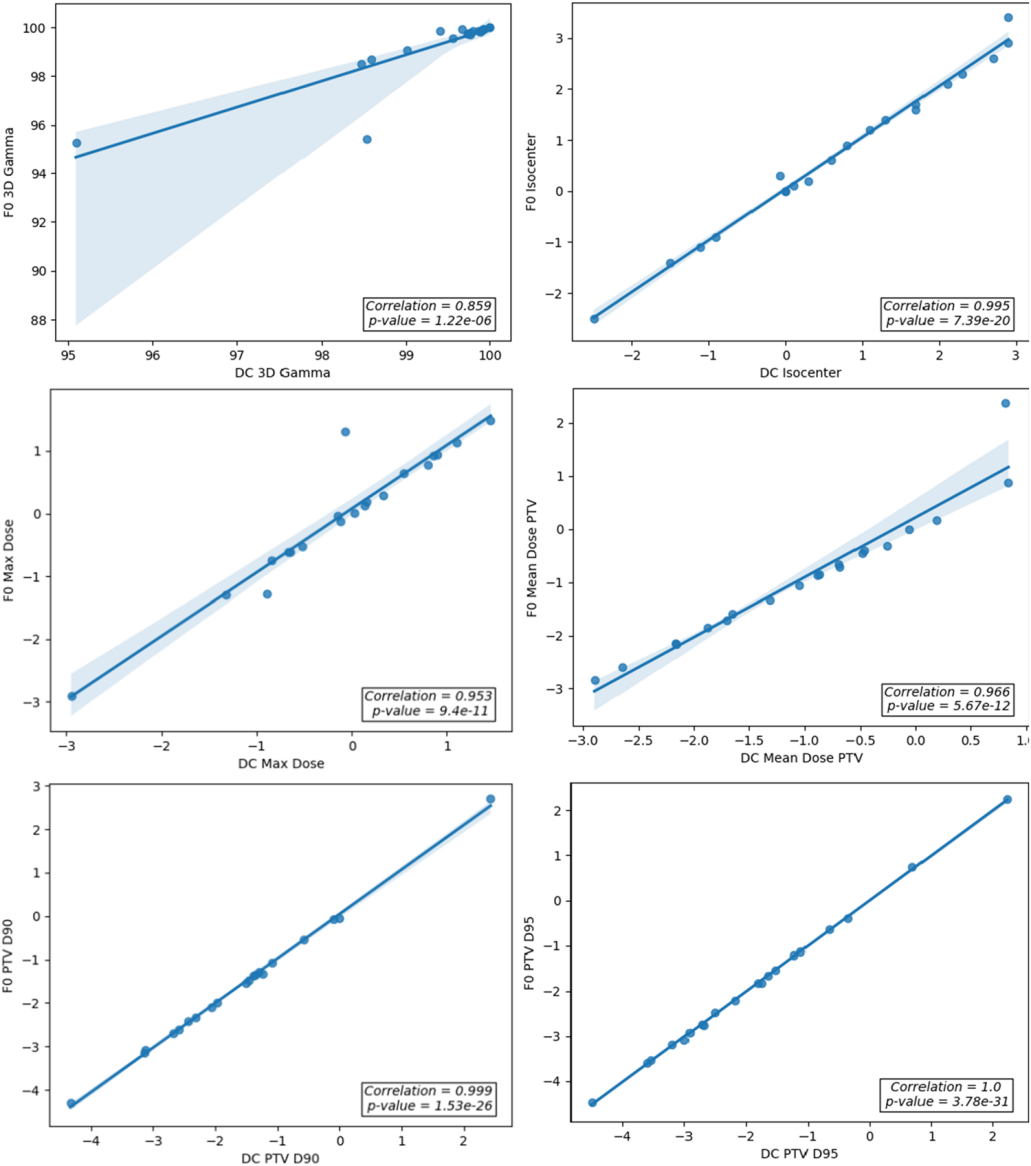
Correlation plots of dosimetric criteria calculated by DC and F0 for AXB lung SBRT plans

Figure 5 illustrates the correlation between DC and F0 for treatment plans calculated utilizing the Acuros XB algorithm.

In both cases the plots indicate that all the chosen criteria are well correlated. The cases planned with the Acuros XB algorithm yielded a mean correlation coefficient for the six criteria defined of 0.962. For the cases planned with the AAA algorithm the average correlation coefficient was 0.991.

The correlation results demonstrated that the same action limits can be used for DC and Fraction 0. The strong correlation also demonstrates that DC results are predictive of Fraction 0 results, which is expected if the plan is delivered correctly. Together, these results led to the determination that DC can be used as the predictive step of the hybrid QA process to determine if a phantom based measurement should be performed for QA, or if delivering the plan for Fraction 0 is sufficient. If a Fraction 0 result were to fail, further investigation would be undertaken including a phantom based QA measurement.

### 3.3 Lung SBRT hybrid QA validation

Table 3 summarizes the results of applying the hybrid QA technique to the lung SBRT plans. There were seven plans flagged for a phantom based QA to be performed when the AAA algorithm was used for the TPS dose calculation. There were 0 flagged when plans were calculated with the Acuros XB algorithm. There was very good agreement with the results of Fraction 0 and the measured SRS MapCheck QA for cases utilizing the Acuros XB algorithm. All plans that passed the DC screening were verified to have passing Fraction 0 trajectory log QA and a passing measurement based IMRT QA.

**TABLE 3:** Validation results of applying the hybrid QA technique to the lung SBRT cases

	AAA		Acuros XB	
	All cases	DC flagged cases (7)	All cases	DC flagged cases
Fraction 0 gamma pass rate	91.1 ± 10.1%	78.9 ± 8.8%	99.3 ± 1.3%	None
SRS MapCheck gamma pass rate	98.3 ± 2.8%	98.6 ± 1.8%	99.1 ± 1.3%	N/A

For the seven AAA plans that were flagged by DC, the Fraction 0 trajectory log QA results were consistent. Correlation of DC and Fraction 0 results combined with the fact that none of the plans failed the SRS MapCheck QA strengthens the case of algorithmic issues with the use of AAA for lung SBRT cases. Based on this, the use of AAA as the TPS dose algorithm for lung cases would make the use of the proposed hybrid QA technique challenging as DC and AAA appear to show larger disagreements and would limit the ability of the technique to identify true treatment delivery related issues. Investigation of the cases that failed when using AAA as the dose algorithm reveal all cases had lesions abutting or close to the chest wall. However, this was not consistent as there were also other cases with lesions abutting the chest wall that passed the DC criteria. More in depth analysis outside the scope of this study would be required to identify causes of the disagreement.

### 3.4 Action limits for MLSRS

**FIGURE 6:**
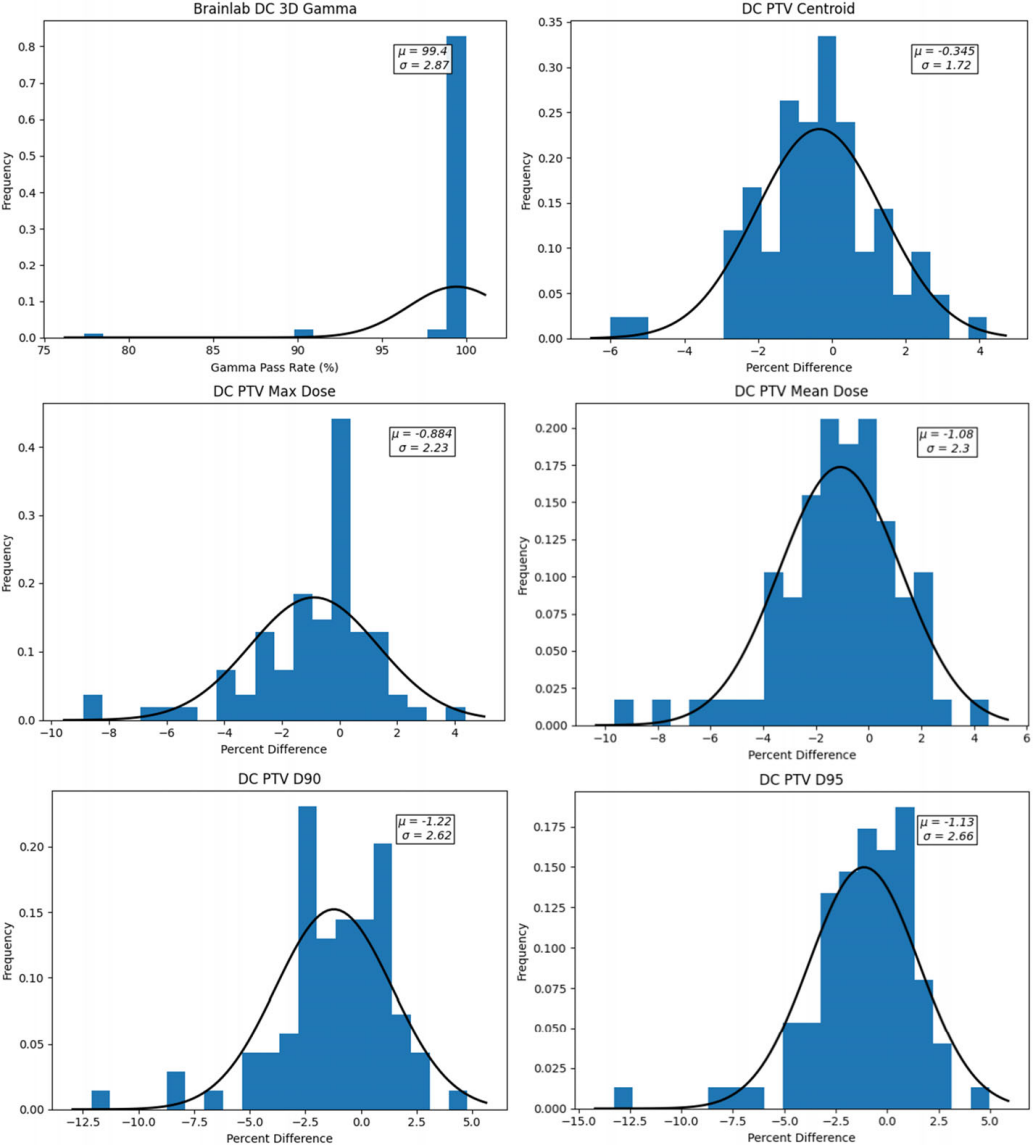
Histogram plots of DC dosimetric criteria agreement for all targets in the MLSRS plans

Figure 6 presents the histogram plots for the recorded DC agreement of the dosimetric criteria for the single isocentre MLSRS cases. These results are summarized in Table 4 and used to calculate the corresponding CL for each of the chosen criteria. The CL action limits calculated were: 4% for the PTV centroid, 5% for the max dose to the PTV, 6% for the mean dose to the PTV; 6% for the D90 to the PTV and 6% for the D95 of the PTV. As with the lung cases, to simplify implementation a uniform CL action limit of 5% was chosen for all criteria.

**TABLE 4:** Summary of the MLSRS dosimetric criteria results and corresponding calculated CL

Criteria	Average	SD	CL
PTV centroid	−0.35	1.72	4%
PTV max dose	−0.88	2.23	5%
PTV mean dose	−1.08	2.3	6%
PTV D90	−1.22	2.62	6%
PTV D95	−1.13	2.66	6%
3D gamma	99.4	2.87	6.2(93.8%)

### 3.5 Correlation of DC and Fraction 0 for MLSRS

**FIGURE 7:**
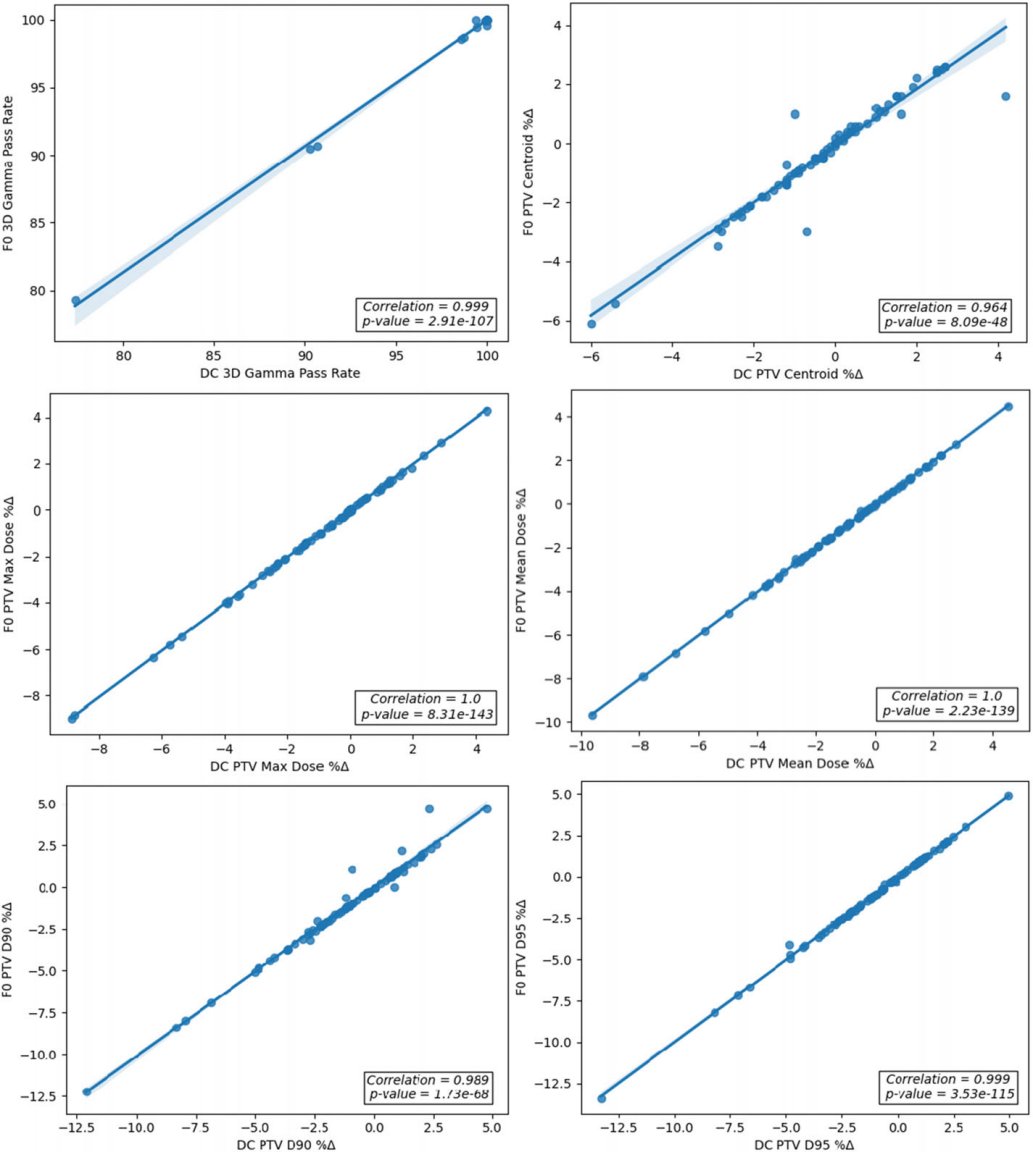
Correlation plots of dosimetric criteria calculated by DC and F0 for MLSRS plans

The correlation of DC and Fraction 0 for all criteria is illustrated in Figure 7.

For all criteria, the agreement of DC and Fraction 0 was very highly correlated, which is consistent with the results of the lung SBRT cases. The average correlation coefficient for all criteria was 0.992. Based on these results, DC can be used as the screening step and the same action limits can be used for DC and F0.

### 3.6 MLSRS hybrid QA validation

Table 5 summarizes the results of applying the hybrid QA technique to the MLSRS plans. There were six lesions flagged for a QA measurement with a phantom. As with the lung cases, all lesions that passed the DC screening were verified to have a passing Fraction 0 trajectory log QA and a passing SRS MapCheck QA. For the flagged lesions, the Fraction 0 and SRS MapCheck QA results were comparable at 95.7% and 98.9% gamma passing rates, respectively. Even though they were flagged for measurement, all corresponding SRS MapCheck QA passed with a gamma pass rate greater than 95%. Investigation of the flagged lesions suggest DC and Fraction 0 disagreement was due to either the volume of the lesion being extremely small, or it was located adjacent to air cavities or bone. Given the passing SRS MapCheck results indicate there wasn't a delivery error, DC and Fraction 0 disagreement is most likely due to limitations of the Elements pencil beam algorithm.

**TABLE 5:** Validation results of applying the hybrid QA technique to the MLSRS cases

	All cases	DC flagged lesions (6)
Fraction 0 gamma pass rate	99.5 ± 2.4%	95.7 ± 8.2%
SRS MapCheck gamma pass rate	99.1 ± 1.1%	98.9 ± 1.7%

## 4 DISCUSSION

The proposed hybrid QA technique can increase efficiency and reduce the amount of measurement based PSQA that would be required. Utilizing the action limits set, for lung SBRT the number of PSQA would be reduced from 22 to 0 if using Acuros XB for the dose calculation, or if AAA is used, a reduction of 22 to 7. What could be considered more impactful is the ability to indicate which targets need to be measured for MLSRS plans. Out of 82 targets, the proposed technique identified six targets that based on the results of the 3D secondary verification should be further investigated with a measurement. Compared to the effort required to measure all targets, or previously reported techniques that pseudo‐randomly select which targets to measure, the proposed technique provides a quantitative method for determining what should be measured and can reduce the number of measurements required.

The use of computational based PSQA in lieu of measurement based IMRT QA has been a topic of debate due to concerns of errors that may not be detected if a measurement is not performed. Conventional measurement based IMRT QA is a single end‐to‐end test that is fundamentally verifying three separate failure modes: accuracy of the dose calculation, plan data transfer and integrity check, and machine errors in the treatment delivery. The proposed hybrid technique still tests these failure modes, but instead relies on separate testing methods which can provide more information for each failure mode. This potentially makes it easier to troubleshoot a plan compared to trying to determine the reason from a single failing measurement based IMRT QA. The first error mode, accuracy of the dose calculation, is verified with the use of DoseCHECK, an independent TPS‐class 3D dose calculation algorithm. Unlike a dose distribution measured on a homogenous phantom, computational QA methods provide DVH information on the actual treatment planning CT that are ultimately relevant to the clinical success and safety of the treatment. Additionally, 3D secondary dose calculation has been demonstrated to have a higher sensitivity in detecting unacceptable plans compared to measurement‐based IMRT QA, as demonstrated in the work presented by Kry et al.^32^

Data transfer failures can be caught by the Fraction 0 step of the proposed technique. Every plan is delivered before patient treatment so the trajectory logs can be analysed. Fraction 0 uses the trajectory logs recorded during plan delivery to reconstruct a plan of what the linear accelerator delivered and calculate that delivered dose on the patient CT. This type of QA could have prevented one of the most serious reported incidents, where MLC control points were absent from an IMRT plan.^33^ A second data transfer failure mode that has been reported in the literature is MLC control points were present but were corrupted during data transfer, causing the leaves to be in the wrong position.^34^ Trajectory logs can detect this error depending on the type of analysis performed. A simple analysis that only examines reported RMS positioning errors would not detect this failure, as the plan was delivered correctly as loaded on the linear accelerator. Fraction 0, however, generates an RT plan from the trajectory logs and compares the delivered dose distribution to the planned, which could detect this type of discrepancy. There have been reported cases in the literature where the position of MLC leaves reported by trajectory logs deviated from the actual leaf position determined from EPID imaging.^35^ This is where detecting the third failure mode becomes critical.

Robust QA of the linear accelerator, independent of PSQA, must be performed to prevent machine errors in delivery of the treatment plan. This includes performing all daily and monthly tests as recommended by TG‐142 and MPPG 8.a. to verify the accelerator is operating nominally.^36^, ^37^ Although the potential always exists for a failure to occur during treatment delivery, monitoring of trends can help detect and rectify issues before they become clinically relevant. The reliance of IMRT plans on the MLC makes the monitoring of their performance crucial, as degradation in their performance can negatively impact the treatment delivery. Malfunctioning motors, loose T‐nuts, and calibration issues have been demonstrated to cause MLC positioning errors of up to 1.5 mm.^35^ These types of errors can affect QA methods that rely on trajectory logs, however, studies have demonstrated that MLC errors up to this magnitude also cannot be detected by common measurement based IMRT QA techniques.^11^, ^12^, ^38^ For these reasons it is critical not to rely on PSQA as the only test of MLC performance. There must be a robust MLC QA program as part of any IMRT program. In our clinic, in addition to weekly picket fence tests, a suite of monthly MLC/VMAT testing and annual MLC tests, we perform the enhanced MLC testing daily as part of the Varian Machine Performance Check (MPC), which verifies leaf positioning and offset to within 1 mm.^39^

One of the purposes of the current study was to characterize agreement of 3D secondary dose verification software for stereotactic treatments. In the case of the lung SBRT plans, it was demonstrated that the level of agreement was dependent on the TPS dose algorithm used. Poorer agreement of DC and AAA was observed compared to DC and Acuros XB. This is consistent with previous studies that have demonstrated better agreement of collapsed cone/superposition algorithms like DC with Acuros XB, compared to AAA.^40^, ^41^, ^42^ Agreement of DC and the MLSRS plans was on average poorer than agreement of DC and the lung SBRT plans calculated with Acuros XB. One possible reason for this is the use of the Pencil Beam dose calculation algorithm within Brainlab Elements, which has limitations in accuracy near tissue heterogeneities compared to a convolution/superposition algorithm. Future work will investigate how the use of the Monte Carlo algorithm within Elements influences agreement with DC. Analysis of the results for both types of stereotactic treatment plans demonstrate how the level of agreement is dependent on the accuracy of the TPS dose algorithm compared to the algorithm used in the 3D secondary verification. Therefore, it is important for each clinic to characterize and understand the expected agreement of their 3D secondary verification software when implementing a QA method like described in the current study.

A growing trend within the medical physics community is the investigation of computational based PSQA methods to reduce the burden of measurement based IMRT QA. Machine learning (ML) based techniques have been a primary area of investigation for computational based methods. Most studies have developed ML models which use plan complexity features or planar dose distributions to predict a gamma pass rate.^43^, ^44^, ^45^, ^46^, ^47^, ^48^ Given the previously discussed low sensitivity of gamma pass rate of conventional IMRT QA measurements to detect errors, it is unclear this method of computational PSQA is robust enough to be able to replace measurement‐based QA. Other ML QA techniques have sought to predict DVH metrics. Lay et al. developed a ML model to predict the trajectory log errors of a plan delivery, from which a DICOM RT plan of the predicted delivery would be generated and used by a monte carlo dose algorithm to calculate the dose and compute DVH agreement.^49^ Wall et al. presented a virtual QA technique that combines independent secondary dose calculation by Mobius3D (Varian) and a ML model to predict the agreement of a point dose ionization chamber measurement, where a 1% tolerance in the predicted agreement was used to determine if a measurement should be performed.^50^ Their use of a computational method to determine whether a measurement should be acquired is similar to the hybrid technique presented in the current study. These studies and the current study demonstrate a trend towards the use of computational based QA to improve efficiency of PSQA.

Future work will investigate applying the technique to other types of radiation therapy plans. Another treatment site that may require the type of characterization of 3D secondary verification performed in the current study is spine SRS/SBRT treatments. Like observed with lung SBRT, bone is another tissue heterogeneity that can cause disagreement between dose calculation algorithms.^28^, ^41^ Stereotactic spine treatments can also be more highly modulated compared to lung SBRT. To more broadly apply the technique to conventionally fractionated treatments throughout the body, a general characterization of larger field treatment plans such as head and neck or prostate with pelvic nodal irradiation could be undertaken.

## 5 CONCLUSION

This study presented a hybrid PSQA technique that uses an independent 3D dose/MU verification software and 95% CL action limits for agreement of dosimetric criteria to determine whether a phantom based QA measurement is needed for Lung SBRT and MLSRS cases. This method has proven to be efficient, for it is part of the normal clinical workflow when verifying clinical treatment plans for these two specific cases. It drastically reduces the number of measurements needed for PSQA which allows the physicist to focus on other tasks within the clinic. Its simplicity makes it easy to implement and provides a uniform methodology to verify the accuracy of the treatment plan and delivery. Although this technique was applied to just MLSRS and lung SBRT cases, the authors believe that it can easily be applied to all VMAT and IMRT cases. This study focused on the use of DoseCheck and Fraction 0 from Sun Nuclear, however, we believe the methodology can be applied using any 3D verification software.

## AUTHOR CONTRIBUTIONS

Garrett C. Baltz and Steven M. Kirsner contributed equally to conception and design of the study, analysis of the data, and writing of the manuscript. Remy Manigold and Richard Seier contributed to data acquisition, analysis, and reviewing of manuscript.

## CONFLICT OF INTEREST

The authors do not have a conflict of interest.

## Data Availability

The data that support the findings of this study are available from the corresponding author upon reasonable request.
